# Real-time segmentation method of billet infrared image based on multi-scale feature fusion

**DOI:** 10.1038/s41598-022-09233-6

**Published:** 2022-04-27

**Authors:** Lixin Zhang, Qingrong Nan, Shengqin Bian, Tao Liu, Zhengguang Xu

**Affiliations:** 1 School of Automation, University of Science and Technology Beijing, Beijing, 100083 China; 2 School of Computer and Communication, University of Science and Technology Beijing, Beijing, 100083 China

**Keywords:** Engineering, Mathematics and computing

## Abstract

Obtaining the surface temperature of billets in heating furnaces has been a hot research in metallurgical industry applications. In order to accurately identify the billet location in infrared images and thus obtain the surface temperature of billets, this paper proposes a real-time segmentation network model based on multi-scale feature fusion to solve the problems of low resolution, low accuracy and slow detection speed of infrared images of traditional target image detection methods. In our method, a dataset with billet infrared images as the experimental object is firstly established, and the proposed network structure adopts multi-scale feature fusion to enhance the information interaction between feature maps at all levels and reduce the information loss during up-sampling by a dense up-sampling strategy. Meanwhile, a lightweight backbone network and deep separable convolution are used to reduce the number of network parameters and speed up the network inference, finally realizing real-time and accurate segmentation of the infrared images of blanks. The highest accuracy of the model in this paper reaches 94.89$\%$. Meanwhile, an inference speed of 80fps is achieved on GTX2080Ti. Compared with the existing mainstream methods, the method in this paper can better meet the real-time and accuracy requirements of industrial production.

## Introduction

Infrared temperature measurement is a mature and dynamic technology that is widely used in many industries and organizations. It is useful for measuring temperature in some typical situations, i.e. when the object to be measured is moving or when a fast response is required^1^. In order to implement a fully automated thermal imaging analysis system for billet temperature, the location of the billet must first be accurately identified in the infrared image of the billet so that the corresponding temperature value can be fitted based on the pixel value. However, infrared imaging suffers from defects such as blurred edges, low contrast and uneven intensity, resulting in a limited segmentation accuracy for the target^2^.

At present, the specialized research for infrared image segmentation processing is significantly less than that for visible images, and is dominated by traditional segmentation algorithms, e.g., Zhou^3^ proposed an infrared image segmentation algorithm based on Otsu and genetic algorithm, but the algorithm has multiple thresholding calculations and the processing is relatively complicated. Ochoa^4^ also used threshold information to segment infrared images and applied it to fault detection of motors, but the segmentation effect for the target was slightly rough. Yin^5^ proposed a dual-even morphological gradient-based edge detection operator to identify and diagnose inferior insulators, and Wang^6^ designed a spiking neural network using the properties of spiking neurons to implement edge detection on infrared images of high-voltage equipment. both of the above methods have made certain contributions to edge detection algorithms for infrared images,but the segmentation effect is poor in cases where the gradient between the target and the background is small. Yu et al.^7^ successfully extracted the target information of IR images based on the region and gradient information of the image, but the algorithm used fuzzy clustering method in extracting the region information, which is more complicated to calculate and difficult to achieve real-time. Most of the above methods use traditional segmentation algorithms in achieving segmentation of infrared targets based on are using low-level features of images (information such as color, texture and shape), which are difficult to apply to billet identification in complex environments in heating furnaces.

In recent years, with the rapid development of computer processing technology, deep learning technology has been widely used in image recognition, semantic segmentation, target detection, and other fields.Different from traditional segmentation methods, the goal of semantic segmentation based on deep learning is to predict the class label of each pixel in the image^8^, and automatically learn features in various scenarios through a large number of sample training, so it has better generalization capabilities and robustness^9^.

To meet the needs of the heating furnace control system and field personnel observation, the real-time and accuracy of the infrared image segmentation of the billet in the furnace is an important indicator of the effectiveness of the segmentation method. Existing methods^10–12^ mainly focus on improving performance. However, achieving real-time performance with low latency is the most critical issue for real applications. At present, there are several ways to improve the speed of network segmentation.Some methods speed up network prediction by reducing the resolution of the input image, such as BiseNet^13^, DFANet^14^, etc., but it will lose some spatial information, especially edge information. Some other methods prune the number of feature channels to reduce the computational consumption, such as ENet^15^, SegNet^16^, etc., and this way will decrease the feature extraction ability of the network. Another common solution is to use fewer downsamples when extracting features, such as ESPNet^17^, ERFNet^18^, etc. These networks have an obvious defect that they cannot achieve sufficient receptive fields. In order to solve the dilemma of real-time semantic segmentation, many improved network architectures have been proposed, such as Spatial Pyramid Pooling (SPP)^19^, Atrous Spatial Pyramid Pooling (ASPP)^12^, and other structures that increase the receptive field.

In summary, the key to real-time semantic segmentation is how to obtain a larger receptive field and restore spatial information while maintaining a smaller computational cost. Therefore, this paper proposes a segmentation method based on multi-scale feature fusion. First, the information interaction between all levels of feature maps is strengthened through multi-scale feature extraction, and a larger receptive field and spatial information recovery are obtained. Secondly, Dense Upsampling Convolution(DUC) strategy is used to retain more information during decoding, to improve the accuracy. Finally, Finally, this paper uses the lightweight backbone network ResNet-18^20^ and deep-wise separable convolution to reduce the computational consumption during feature fusion. Experiments have proved that the network proposed in this paper performs well in segmentation accuracy and efficiency, and achieves a detection speed of 80fps and 94.89$\%$ mIoU on GTX2080Ti.

## Methods

**Figure 1:**
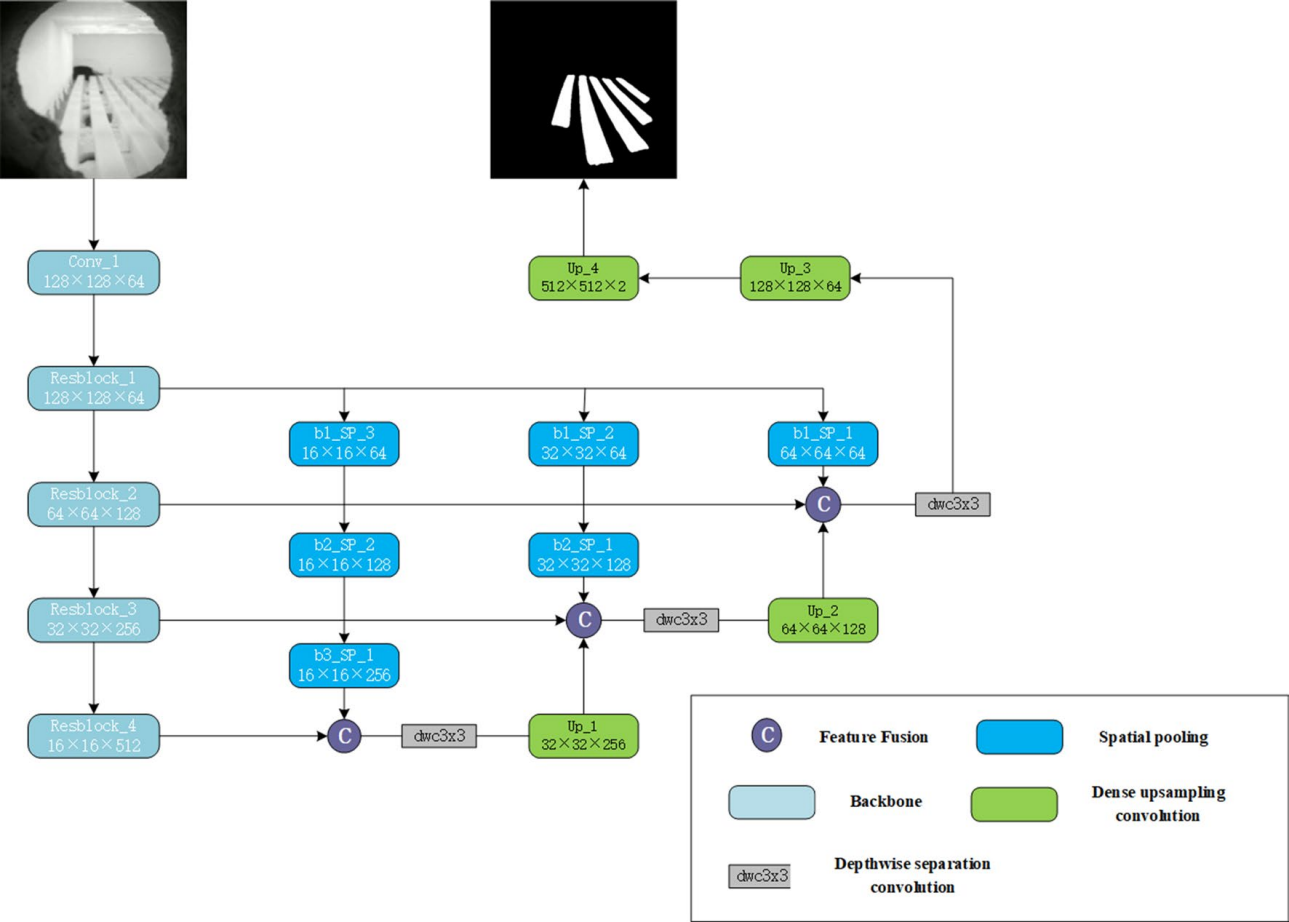
The network structure of the method in this paper.

In this paper, we propose a real-time segmentation method for infrared images based on a lightweight network, ResNet-18, for multi-scale feature fusion. The overall structure of this network is shown in Fig. 1. The specific steps are as follows.

- Improve the information interaction between the feature maps at all levels of the network through multi-scale feature fusion, and strengthen the multi-scale expression ability of the network;
- Use Dense Upsampling Convolution(DUC) strategy to reduce information loss, retain more image feature information, and improve segmentation accuracy;
- Use hole convolution and asymmetric convolution to obtain a larger image receptive field, more fully integrate the context information of the image, and further improve the accuracy of segmentation;
- Reduce the number of network parameters through lightweight backbone network and depth-wise separable convolution, speed up model inference, and realize real-time segmentation.

### Feature extraction module

**Figure 2:**
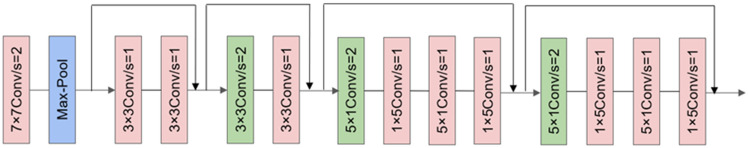
The network structure of the feature extraction module.

The feature extraction module of the method in this paper is shown in Fig. 2. Its main structure uses a lightweight network ResNet-18^20^, which has four different residual blocks, and each residual block contains two 3 $\times$ 3 convolutions and one skip connection, ResNet-18^20^ solves the problem of gradient dispersion and gradient explosion in deep convolutional networks by using the residual structure, but it still has some drawbacks such as small receptive field and single feature extraction size.

In order to further reduce the parameters, expand the perceptual field and improve the segmentation accuracy, in this paper, asymmetric convolution^21^ and dilated convolution^22^ are applied in Resblock$\_$3 and Resblock$\_$4. Asymmetric convolution is the decomposition of a standard two-dimensional convolution into two one-dimensional convolutions, i.e., the traditional n$\times$n convolution is decomposed into n$\times$1 and 1 $\times$ n convolutions. This approach has two advantages^23^:increasing the nonlinearity of the network and improving the discriminative power of the network; and reducing the network parameters and computational effort. Dilated convolution can be considered as convolution with holes. The basic principle is to insert a hole (i.e. a pixel with a value of 0) between each pixel of the normal convolution kernel, increasing the field of view of the network without increasing the number of network parameters.

### Muti-scale feature fusion

In semantic segmentation, to obtain a larger receptive field and a smaller computational cost, a down-sampling operation is required^24^. However, this will lose a large amount of spatial information, especially information related to edges. Based on the above analysis, this paper proposes a method of fusing multi-scale feature information to improve the multi-scale expression ability of the network. Specifically, after each residual block of the backbone network, pooling operations of different scales are performed to generate feature maps of different scales. In order to expand the receptive field and extract richer feature information, the pooling operation is used as the step size $s = 2^j$, and the convolution kernel size is $k = 2 \times s+1 = 2^ {j+1} +1, j \in [1,2,3]$, j is the pooling level. For the feature map of $B \in {\mathbb {B}} ^ {C \times H \times W}$,after the pooling operation, the size of the feature map is $O^{j} \in {\mathbb {B}}^{C \times \frac{H}{2^{j}}} \times \frac{W}{2^j}$, (H, W)is the height and width of the feature map, and C is the number of channels in the feature map. We perform a 3-level pooling operation on the feature map output by Resblock_1, perform 2-level and 1-level pooling operations on Resblock_2 and Resblcok_3 respectively, and finally aggregate the feature maps with the same resolution. Due to a large number of channels after aggregation, the depth-wise separation convolution^25^ is used for fusion to reduce the amount of calculation for fusion. By merging the feature information extracted from different layers in the backbone network, the interaction between low-level spatial information and high-level semantic information is strengthened, thereby improving the accuracy of the network.

### Dense up-sampling convolution

There are three general up-sampling methods for networks under the semantic segmentation task: bilinear interpolation, de-pooling operation, and deconvolution operation. Among them, bilinear interpolation is not learnable, and detailed information will be lost, while the de-pooling operation and deconvolution operation will lose part of the information, which affects the segmentation accuracy. Based on the above situation, Panqu Wang et al.^26^proposed dense up-sampling convolution (DUC), which uses channel dimensions to make up for the loss in length and width. Specifically, the input image size is set to H$\times$W, the feature map after the feature extraction stage is $h \times w \times c (h=H/d,w=W/d)$, and convolution is applied to this feature map. The resulting output feature map is $h \times w \times (d^2 \times L)$, where d is the down-sampling multiple and L is the total number of categories of the segmentation task, and finally through pixel shuffle to H$\times$W$\times$L.

**Figure 3:**
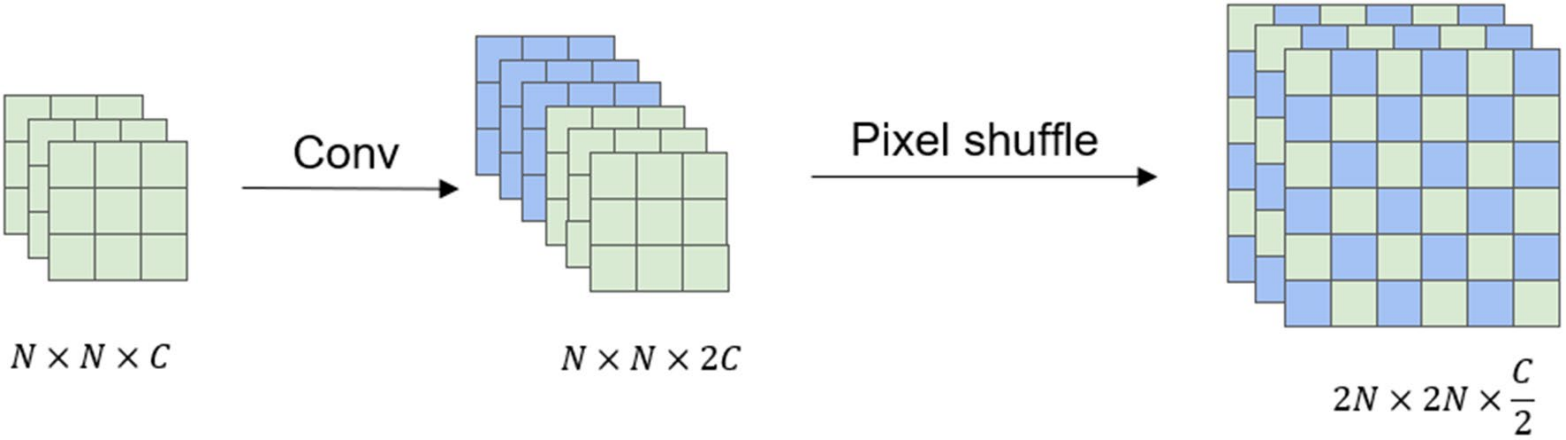
Dense up-sampling module structure.

In this paper, dense up-sampling convolution is used in the decoding part of the network structure, and the up-sampling task is allocated to each layer to fuse the information of different layers. The specific structure is shown in Fig. 3. When sampling on each layer, the feature graph is only restored to the size of the previous layer, and the information of each layer is merged with a small computational consumption, which further avoids the loss of information during decoding, and further improves the segmentation accuracy.

**Table 1:** Experiment results of the effectiveness of the network structure.

Experiment	Asymmetric + dilated convolution	Muti-scale feature fusion	Dense up-sampling	mIoU $(\%)$	BIoU $(\%)$
1				93.68	55.85
2	$\checkmark$			93.91	56.17
3		$\checkmark$		94.41	57.05
4			$\checkmark$	94.08	56.44
5	$\checkmark$	$\checkmark$		94.46	57.12
6	$\checkmark$		$\checkmark$	94.25	56.89
7		$\checkmark$	$\checkmark$	94.74	57.53
8	$\checkmark$	$\checkmark$	$\checkmark$	94.89	57.71

**Table 2:** Speed and accuracy analysis.

Model	GFLOPs	Parameters	Frame (fps)	mIoU $$(\%)$$	PA $$(\%)$$	BIoU^29^$$^ (\%)$$
U-shape^27^	6.9	18.39M	83.80	93.68	98.84	55.85
MobileNet^28^	3.9	22.09M	121.41	93.54	98.61	52.08
ENet^15^	3.56	0.4M	26.57	93.69	98.63	46.07
ESPNet^17^	0.79	0.264M	40.26	94.28	98.77	52.47
ERFNet^18^	6.4	2.06M	46.25	94.74	98.89	57.58
BiseNet^13^	10.8	12.41M	125.98	93.46	98.60	46.08
Ours	8.6	20.23M	79.83	94.89	98.91	57.70

## Experiment

### Data set preparation

In this study, the infrared images of billets at the exit of the heating furnace are used to intercept frames from the video surveillance data, and a total of 5000 images of billets in different states are intercepted, and a $512 \times 512$ area in the center of the image (the area contains most of the information of the billets) is intercepted. The intercepted images are annotated to obtain the target images, and the training set, validation set and test set are divided according to the ratio of 7:2:1.

### Experiment method

In order to verify the effectiveness of the proposed network, we conducted detailed experiments on an experimental platform configured with GTX2080Ti, cuda 10.0, cudnn 7, and pytorch 1.1.0. the model was configured with an Adam optimizer, a batchsize size of 4, an initial learning rate of 1e−4, and 2000 epochs of training rounds. in order to To enhance the robustness of the network, we used data enhancement methods such as random flip, random Gaussian blur, random brightness adjustment, and set random values of [0.5, 2] as the image scale for scaling.

#### Effectiveness of the network structure

The method in this paper consists of three main parts: a multiscale feature fusion module, a dense upsampling convolution module, asymmetric convolution, and dilated convolution. In order to verify the effectiveness of the network structure proposed in this paper, we conducted detailed comparison experiments. The experimental results are shown in Table 1. from the experimental results, it can be seen that each optimization strategy proposed in this paper has certain superiority, among which Muti-scale Feature Fusion has obvious effect on the prediction of targets and has significant improvement on the prediction of target boundary regions. In this experiment, the basic network is a traditional U-Shape^27^ structured network.

#### Comparison of network inference speed and accuracy

**Figure 4:**
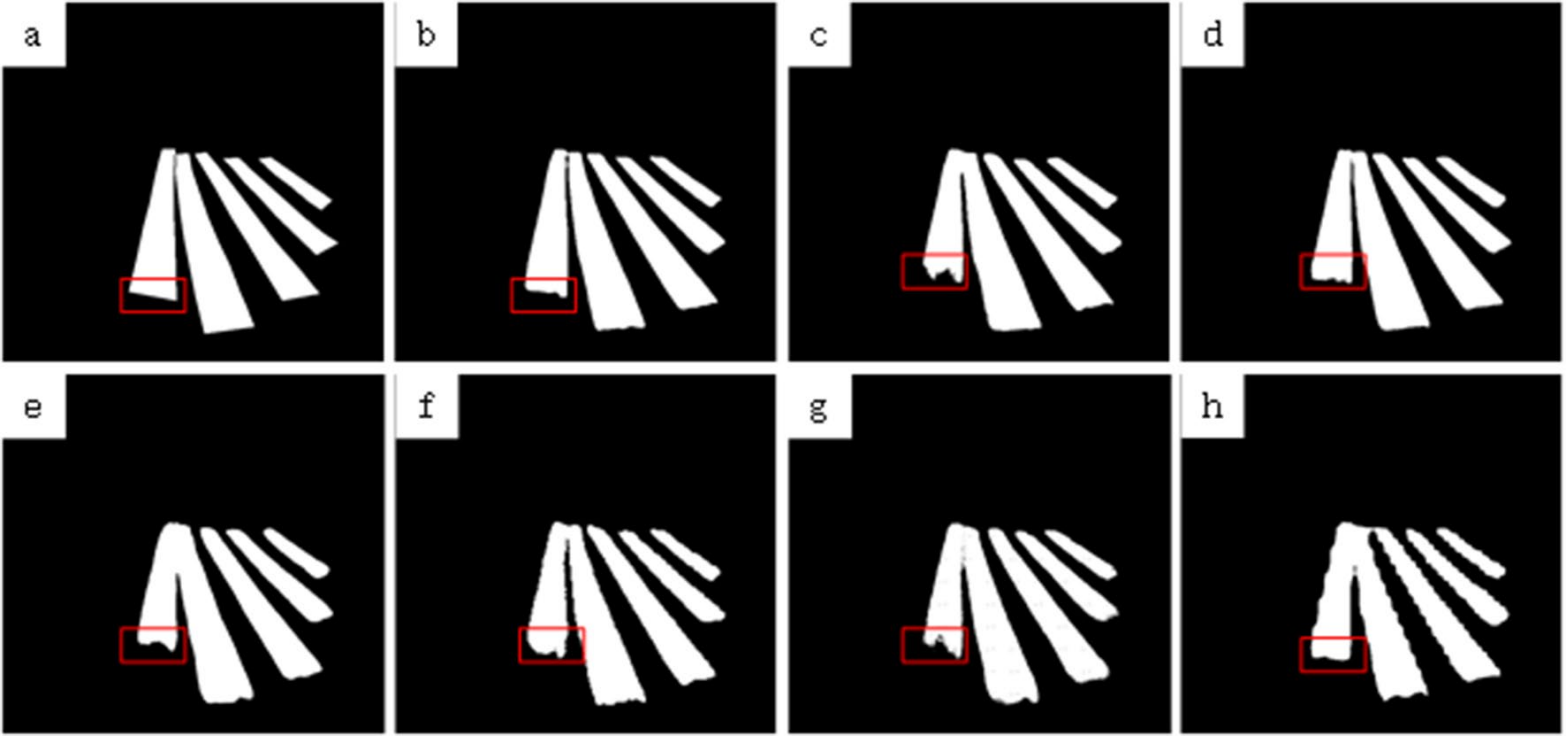
Comparison of image segmentation results by different methods. (**a**) Ground truth, (**b**) ours model, (**c**) U-shape, (**d**) ERFNet, (**e**) ENet, (**f)** EspNet, (**g**) mobileNet, (**h**) BiseNet.

**Figure 5:**
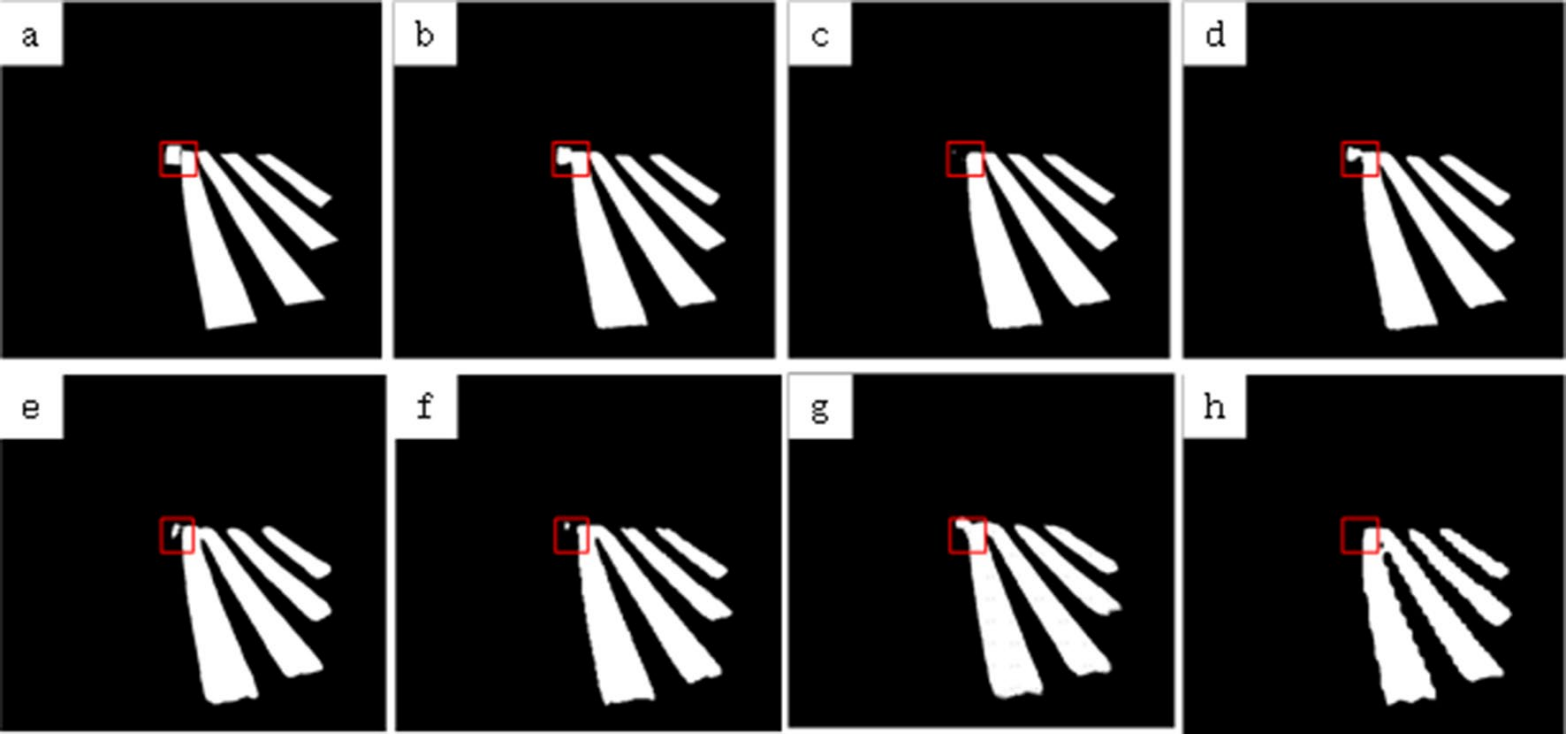
Other comparison.

To further verify the segmentation performance, some commonly used real-time segmentation networks are selected for comparison experiments in this paper, including ENet^15^, EspNet^17^, ErfNet^18^ and BiseNet^13^. in addition, to verify whether the selection of backbone network is reasonable in this paper, the backbone network is replaced with MobileNet^28^ for comparison experiments under the same training parameters. The experimental results are shown in Table 2, among which, BiseNet^13^ has the fastest prediction rate, but its prediction effect for the target is poor, especially in the boundary area of the target, ENet^15^ also has poor prediction effect for the boundary area of the target, and ErfNet^18^ has the closest prediction result with the method of this paper, but the processing efficiency is less than half of the method of this paper. Figures 4 and 5 show some of the results of the comparison experiments.

## Conclusions

In this paper, we propose a real-time semantic segmentation method, based on the proposed multi-scale feature fusion strategy and dense up-sampling convolution strategy, and apply it to the segmentation of the infrared image of the heating furnace billet. Our network can achieve excellent segmentation accuracy and efficiency, thereby greatly improving the segmentation effect of billets. Finally, by comparing with other existing segmentation methods, the results clearly show that our method has greater advantages in terms of segmentation accuracy and efficiency, meeting the temperature measurement requirements of real-time and segmentation accuracy.

## References

[1] Merchant J (2008). Infrared Temperature Measurement Theory and Application.

[2] Wan M (2018). A level set method for infrared image segmentation using global and local information. Remote Sens..

[3] Zhou, S., Yang, P. & Xie, W. Infrared image segmentation based on otsu and genetic algorithm. in *2011 International Conference on Multimedia Technology*. 5421–5424. (IEEE, 2011).

[4] Resendiz-Ochoa E, Osornio-Rios RA, Benitez-Rangel JP, Romero-Troncoso RDJ, Morales-Hernandez LA (2018). Induction motor failure analysis: An automatic methodology based on infrared imaging. IEEE Access.

[5] Yin J, Lu Y, Gong Z, Jiang Y, Yao J (2019). Edge detection of high-voltage porcelain insulators in infrared image using dual parity morphological gradients. IEEE Access.

[6] Wang B, Chen L, Zhang Z (2019). A novel method on the edge detection of infrared image. Optik.

[7] Xiao Y, Zijie Z (2020). Infrared image extraction algorithm based on adaptive growth immune field. Neural Process. Lett..

[8] Han C, Duan Y, Tao X, Lu J (2019). Dense convolutional networks for semantic segmentation. IEEE Access.

[9] Li Q, Fan S, Chen C (2019). An intelligent segmentation and diagnosis method for diabetic retinopathy based on improved u-net network. J. Med. Syst..

[10] Long, J., Shelhamer, E. & Darrell, T. Fully convolutional networks for semantic segmentation. in *Proceedings of the IEEE Conference on Computer Vision and Pattern Recognition*. 3431–3440. (2015). 10.1109/TPAMI.2016.2572683 27244717

[11] Ronneberger, O., Fischer, P. & Brox, T. U-net: Convolutional networks for biomedical image segmentation. in *International Conference on Medical Image Computing and Computer-Assisted Intervention*. 234–241. (Springer, 2015).

[12] Chen, L.-C., Zhu, Y., Papandreou, G., Schroff, F. & Adam, H. Encoder-decoder with atrous separable convolution for semantic image segmentation. in *Proceedings of the European Conference on Computer Vision (ECCV)*. 801–818. (2018).

[13] Yu, C. *et al.* Bisenet: Bilateral segmentation network for real-time semantic segmentation. in *Proceedings of the European Conference on Computer Vision (ECCV)*. 325–341. (2018).

[14] Li, H., Xiong, P., Fan, H. & Sun, J. Dfanet: Deep feature aggregation for real-time semantic segmentation. in *Proceedings of the IEEE/CVF Conference on Computer Vision and Pattern Recognition*. 9522–9531. (2019).

[15] Paszke, A., Chaurasia, A., Kim, S. & Culurciello, E. Enet: A deep neural network architecture for real-time semantic segmentation. *arXiv preprint*: arXiv:1606.02147 (2016).

[16] Badrinarayanan V, Kendall A, Cipolla R (2017). Segnet: A deep convolutional encoder-decoder architecture for image segmentation. IEEE Trans. Pattern Anal. Mach. Intell..

[17] Mehta, S., Rastegari, M., Caspi, A., Shapiro, L. & Hajishirzi, H. Espnet: Efficient spatial pyramid of dilated convolutions for semantic segmentation. in *Proceedings of the European Conference on Computer Vision (ECCV)*. 552–568. (2018).

[18] Romera E, Alvarez JM, Bergasa LM, Arroyo R (2017). Erfnet: Efficient residual factorized convnet for real-time semantic segmentation. IEEE Trans. Intell. Transp. Syst..

[19] Zhao, H., Shi, J., Qi, X., Wang, X. & Jia, J. Pyramid scene parsing network. in *Proceedings of the IEEE Conference on Computer Vision and Pattern Recognition*. 2881–2890. (2017).

[20] He, K., Zhang, X., Ren, S. & Sun, J. Deep residual learning for image recognition. in *Proceedings of the IEEE Conference on Computer Vision and Pattern Recognition*. 770–778. (2016).

[21] Szegedy, C., Vanhoucke, V., Ioffe, S., Shlens, J. & Wojna, Z. Rethinking the inception architecture for computer vision. in *Proceedings of the IEEE Conference on Computer Vision and Pattern Recognition*. 2818–2826. (2016).

[22] Yu, F. & Koltun, V. Multi-scale context aggregation by dilated convolutions. *arXiv preprint* arXiv:1511.07122 (2015).

[23] Alvarez, J. & Petersson, L. Decomposeme: Simplifying convnets for end-to-end learning. *arXiv preprint* arXiv:1606.05426 (2016).

[24] Fan L, Kong H, Wang W-C, Yan J (2018). Semantic segmentation with global encoding and dilated decoder in street scenes. IEEE Access.

[25] Chollet, F. Xception: Deep learning with depthwise separable convolutions. in *Proceedings of the IEEE Conference on Computer Vision and Pattern Recognition*. 1251–1258. (2017).

[26] Wang, P. *et al.* Understanding convolution for semantic segmentation. in *2018 IEEE Winter Conference on Applications of Computer Vision (WACV)*. 1451–1460. (IEEE, 2018).

[27] Chaurasia, A. & Culurciello, E. Linknet: Exploiting encoder representations for efficient semantic segmentation. in *2017 IEEE Visual Communications and Image Processing (VCIP)*. 1–4. (IEEE, 2017).

[28] Howard, A. G. *et al.* Mobilenets: Efficient convolutional neural networks for mobile vision applications. *arXiv preprint* arXiv:1704.04861 (2017).

[29] Cheng B, Girshick R, Dollár P. *et al.* Boundary IoU: Improving object-centric image segmentation evaluation[C]//Proceedings of the IEEE/CVF Conference on Computer Vision and Pattern Recognition. **2021**,15334–15342 10.1109/CVPR46437.2021.01508 (2021).

